# Enhanced Diagnostic Accuracy for Dental Caries and Anomalies in Panoramic Radiographs Using a Custom Deep Learning Model

**DOI:** 10.7759/cureus.67315

**Published:** 2024-08-20

**Authors:** Suvarna Bhat, Gajanan Birajdar, Mukesh Patil

**Affiliations:** 1 Electronics Engineering, Ramrao Adik Institute of Technology, DY Patil University, Navi Mumbai, IND; 2 Computer Engineering, Vidyalankar Institute of Technology, Mumbai, IND; 3 Electronics and Telecommunication Engineering, Ramrao Adik Institute of Technology, DY Patil University, Navi Mumbai, IND

**Keywords:** image pre-processing, dental caries, panoramic dental radiographs, deep learning, medical image processing

## Abstract

Background

Dental caries is one of the most prevalent conditions in dentistry worldwide. Early identification and classification of dental caries are essential for effective prevention and treatment. Panoramic dental radiographs are commonly used to screen for overall oral health, including dental caries and tooth anomalies. However, manual interpretation of these radiographs can be time-consuming and prone to human error. Therefore, an automated classification system could help streamline diagnostic workflows and provide timely insights for clinicians.

Methods

This article presents a deep learning-based, custom-built model for the binary classification of panoramic dental radiographs. The use of histogram equalization and filtering methods as preprocessing techniques effectively addresses issues related to irregular illumination and contrast in dental radiographs, enhancing overall image quality. By incorporating three separate panoramic dental radiograph datasets, the model benefits from a diverse dataset that improves its training and evaluation process across a wide range of caries and abnormalities.

Results

The dental radiograph analysis model is designed for binary classification to detect the presence of dental caries, restorations, and periapical region abnormalities, achieving accuracies of 97.01%, 81.63%, and 77.53%, respectively.

Conclusions

The proposed algorithm extracts discriminative features from dental radiographs, detecting subtle patterns indicative of tooth caries, restorations, and region-based abnormalities. Automating this classification could assist dentists in the early detection of caries and anomalies, aid in treatment planning, and enhance the monitoring of dental diseases, ultimately improving and promoting patients’ oral healthcare.

## Introduction

Tooth decay is one of the most common dental problems worldwide. The term “cavity” in dentistry corresponds to a frequent type of dental decay [1]. Dental caries result from the interaction of oral bacteria and fermentable carbohydrates, which produce acids that harm teeth over time. Early childhood caries is a huge global oral health problem that affects children. Multiple risk factors, including irregular dental checkups, insufficient oral care, sugary diets, and pathogenic germs, contribute to this condition [2]. The WHO estimates that in the majority of nations, 60-90% of children suffer from early childhood caries. Up to 70% of cases occur frequently in developing countries [3]. An early and accurate diagnosis of caries can lead to the implementation of appropriate preventive measures and conservative measures, which can save healthcare costs [4].

X-rays play a crucial role in diagnosing dental caries and impacting supernumerary teeth in dentistry [5]. Despite being the most widely used tool, dental radiography is a subjective way to assess oral health in dental practice. This subjectivity is influenced by several aspects, such as viewing settings, expert standards, dental radiograph quality, and examination duration [6]. The first diagnosis of caries, or whether caries exist or not, often varies significantly between the opinions of different human dentists. Therefore, the development of automated systems for diagnosing oral health is required to reduce the subjectivity associated with human examiners and allow for the early detection of oral health problems.

The literature describes a variety of strategies for analyzing oral health. These techniques include machine and deep learning (DL)-based approaches as well as traditional image processing-based methods. A significant amount of research has been published since 2019 that has discussed how to classify oral health [7]. DL, a subfield of machine learning (ML), serves as the cornerstone for modern artificial intelligence medical imaging systems. DL’s use of high-capacity neural networks trained on datasets sets it apart from prior ML techniques, enabling automatic feature extraction [8]. They can construct classification systems to find repeating patterns and features in massive datasets. Various DL algorithms for determining oral health and dental caries have been reported in the literature. Utilizing a deep convolutional neural network (CNN) built on the GoogleNet Inception v3 architecture, Lee et al. [9] achieved 89% accuracy for premolar caries detection. In a pilot study that investigated caries identified in third molars, Vinayahalingam et al. [10] tested a collection of 100 cropped panoramic dental radiographs using trained MobileNetV2. The model achieved 90% area under the curve. A modified ResNet backbone was presented by Li et al. [11] in order to detect caries on periapical radiographs. With an F1 score of 0.8290, the model successfully identified the dental caries. Karakuş et al. [12] have used YOLOv8 for the detection of different caries, i.e., occlusal, interproximal, and secondary. Their experiment achieved an average sensitivity of 0.932.

The current research on dental caries detection emphasizes the necessity of early and accurate detection in order to avoid progression and provide appropriate treatment. Several methods have been investigated, including visual examination, radiography, and more recent technologies such as AI and ML approaches, which are being used in caries detection systems to improve accuracy and automate the process. In general, ongoing studies aspire to develop comprehensive, noninvasive, and cost-effective methods for improving the identification and management of dental caries. Also, DL techniques are transforming dental healthcare, benefiting clinicians and the system. CNNs have a significant impact on medical image processing. They are highly successful in image classification and play a crucial role in DL. CNN analyzes each pixel in the X-ray by first dividing it into numerous matrices. They employed random selection and grid search to identify certain patterns, such as nodules [13].

Existing research has studied the application of DL in oral health detection, but there are several gaps, like the fact that many DL models are not specifically designed for panoramic dental radiographs. They tend to depend on pre-trained models designed for general medical imaging or other domains, which may not adequately capture the distinctive characteristics of dental radiographs. The existing model’s diagnostic accuracy, while promising, is not consistently superior. The literature study shows that researchers are still striving for a more reliable model to categorize healthy teeth using dental radiographs. Previous research indicates that pre-trained models require significant training time due to their multiple convolutional layers [14]. Pretrained models performed less successfully due to negative transfer learning and overfitting generated by weights from the ImageNet dataset [15]. There is a need for more automated, user-friendly diagnostic tools that can easily fit into clinical workflow, decreasing clinical workflow and reducing dentists’ workload while preserving or improving diagnostic accuracy.

To overcome the limits of the current screening methods for dental caries in panoramic radiographs, this study proposes developing a custom-built CNN-based model that is created for the binary classification of dental panoramic radiographs. This experimental analysis employs three separate datasets, namely the Universidade Federal da Bahia-Universidade Estadual de Santa Cruz (UFBA-UESC), Tuft, and pediatric dental radiograph datasets. Two classes from each set of data were identified and binary classified. For the pediatric dental radiograph dataset, the two classes were “teeth with caries” and “healthy teeth.” For the UFBA-UESC datasets, the two classes were “teeth with restoration” and “teeth without restoration." Finally, for the Tuft dataset, the two classes of “normal teeth” and “teeth with periapical region-based abnormality” were identified. Additionally, the study uses various assessment methods to demonstrate the superior performance of the suggested model. The model outperforms all of the refined pre-trained models in every performance indicator. The major contributions of the study are the following: (1) A custom-built model is intended for binary classification of panoramic dental radiographs, with the aim of enhancing computing efficiency while maintaining high accuracy. This makes the model suitable for deployment in resource-constrained environments. (2) The use of the preprocessing technique efficiently tackles the issues of irregular illumination and poor contrast in dental panoramic radiographs, resulting in improved image quality. (3) Three different panoramic dental radiographs are diversified datasets that enhance the model’s training and evaluation process with a wide range of caries and dental abnormality instances. (4) The technique employs binary classification to detect the presence of dental caries, restoration, and periapical region-based abnormalities, thus improving detection capabilities.

## Materials and methods

**Figure 1:**
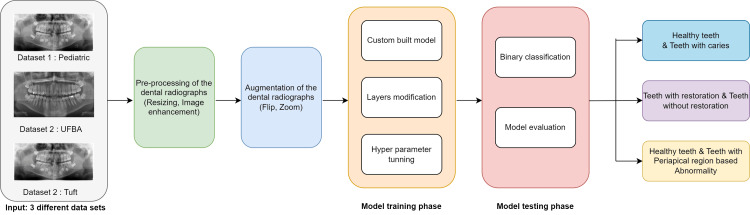
Custom-built DRA model for panoramic dental radiograph classification DRA, dental radiograph analysis

This section describes the custom-built dental radiograph analysis (DRA) model, the proposed framework, and data preprocessing techniques. Figure 1 shows an overview of the proposed methodology. The block diagram depicts the panoramic dental radiograph classification model. The obtained dental radiograph dataset is next subjected to preprocessing steps such as scaling and image-enhancing techniques. The dental radiograph data is then augmented, and the clinical datasets are utilized to train and evaluate the model.

We adopted a DL CNN model specifically for dental caries in panoramic radiographs. Our DRA model, which is a DL-based model, was created to handle the distinct features of dental radiographs. The DRA model is a custom-built 28-layer model. A research study was conducted to better understand the characteristics of each layer and its parameters. The model’s performance is evaluated based on accuracy, precision, recall, F1 score, and confusion matrix for binary classification.

Dataset

Three panoramic dental radiograph datasets were used for this research work; a brief description of the pediatric, UFBA-UESC, and Tuft dental panoramic dental datasets is provided below. Zhang et al. [16] collected dental panoramic radiographs and cases from 106 pediatric patients, ages two to 13. A total of six dental experts were involved in the design and implementation of the annotation work. They used labelMe, an image-annotating software. The authors published their first set of pediatric panoramic oral X-rays to identify dental anomalies, caries segmentation, and annotation detection. Additionally, 93 panoramic radiographs have also been compiled by the authors as supplemental information. This assisted in creating the two classes in the dataset.

There is another publicly accessible UFBA-UESC dental dataset that has 1,500 panoramic dental radiographs divided into 10 distinct groups. According to Silva et al. [17], these radiographs were obtained from the Diagnostic Imaging Center of the Southwest State Universidade Estadual do Sudoeste da Bahia (UESB), city of Vitória da Conquista, in the state of Bahia, Brazil, and the images were categorized based on the tooth structure. For this research study, two categories for binary classification, i.e., “teeth with restoration” and “teeth without restoration,” are being considered.

**Figure 2:**
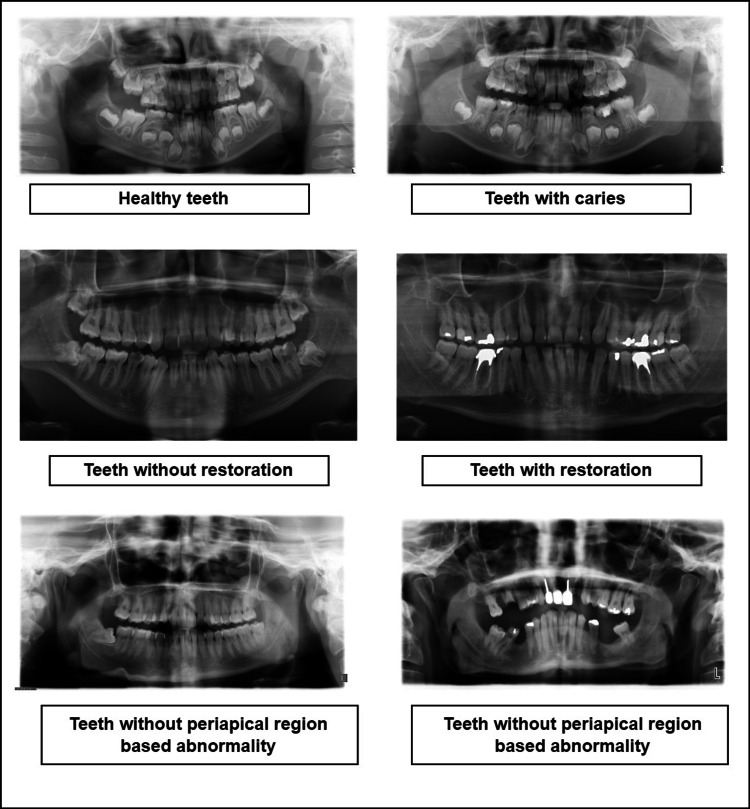
Sample dental radiographs used in the experimental analysis of the study

Tuft’s dental data set was published in December 2021, and it is accessible upon request. At the research center of Tuft University, the authors obtained radiographs. The data collection includes labeled tooth masks and a total of 1,000 panoramic dental radiographs. An expert from Tufts University’s School of Dental Medicine annotated each of these radiographs. Anatomical location, peripheral characteristics, radio density, impacts on the surrounding structure, and abnormality category were the five levels used to classify 1,000 radiography images [18]. For this research study, two classes from this dental panoramic radiograph dataset, namely “periapical region-based anomaly” and “normal condition,” are being considered. Figure 2 shows sample dental radiographs with binary classes from all the datasets mentioned above.

Preprocessing

Larger and more varied training datasets have proved to be quite beneficial for recent advances in DL. However, privacy issues and labeling costs make it difficult to collect large datasets for medical imaging. Without actually collecting new samples, data augmentation enables the researchers to greatly increase the range of datasets available for study [1]. Simple yet remarkable modifications like cropping, padding, and flipping, as well as complicated generative models, are examples of data augmentation approaches [19].

**Figure 3:**
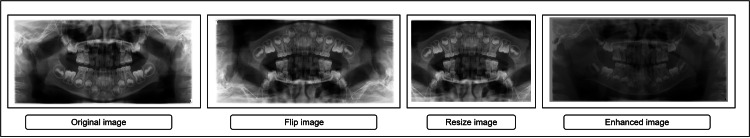
Sample of the data augmentation images

An image data augmentation approach is applied to the training dataset to address dataset limitations. This strategy is shown to enhance the efficiency of the model and its generalization abilities when evaluated on new images. A data augmentation package to rotate, flip, and resize the image to the same scale is used. Figure 3 displays the image style used for data augmentation. After the use of augmentation techniques, all three datasets are balanced, which indicates 200 radiographs per dataset.

**Figure 4:**
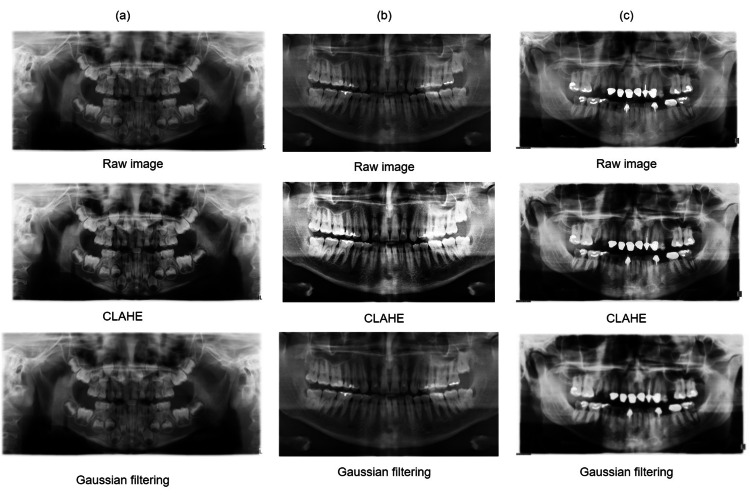
Preprocessed sample dataset

Medical images often pose interpretative challenges due to factors such as inherent noise, uneven illumination, blurriness, low contrast, and incorrect exposure, all of which significantly impact image quality and diagnostic accuracy. To address these issues, image preprocessing techniques are essential [20]. In this experimental analysis, a series of preprocessing methods were applied. Initially, the contrast-limited adaptive histogram equalization technique was employed, known for its effectiveness in enhancing local details in medical images, which facilitates feature detection. Additionally, Gaussian filters, widely used in medical imaging for noise reduction and edge preservation, were applied. Figure 4 illustrates the application of these methods across the three different datasets used in this study.

Framework

**Figure 5:**
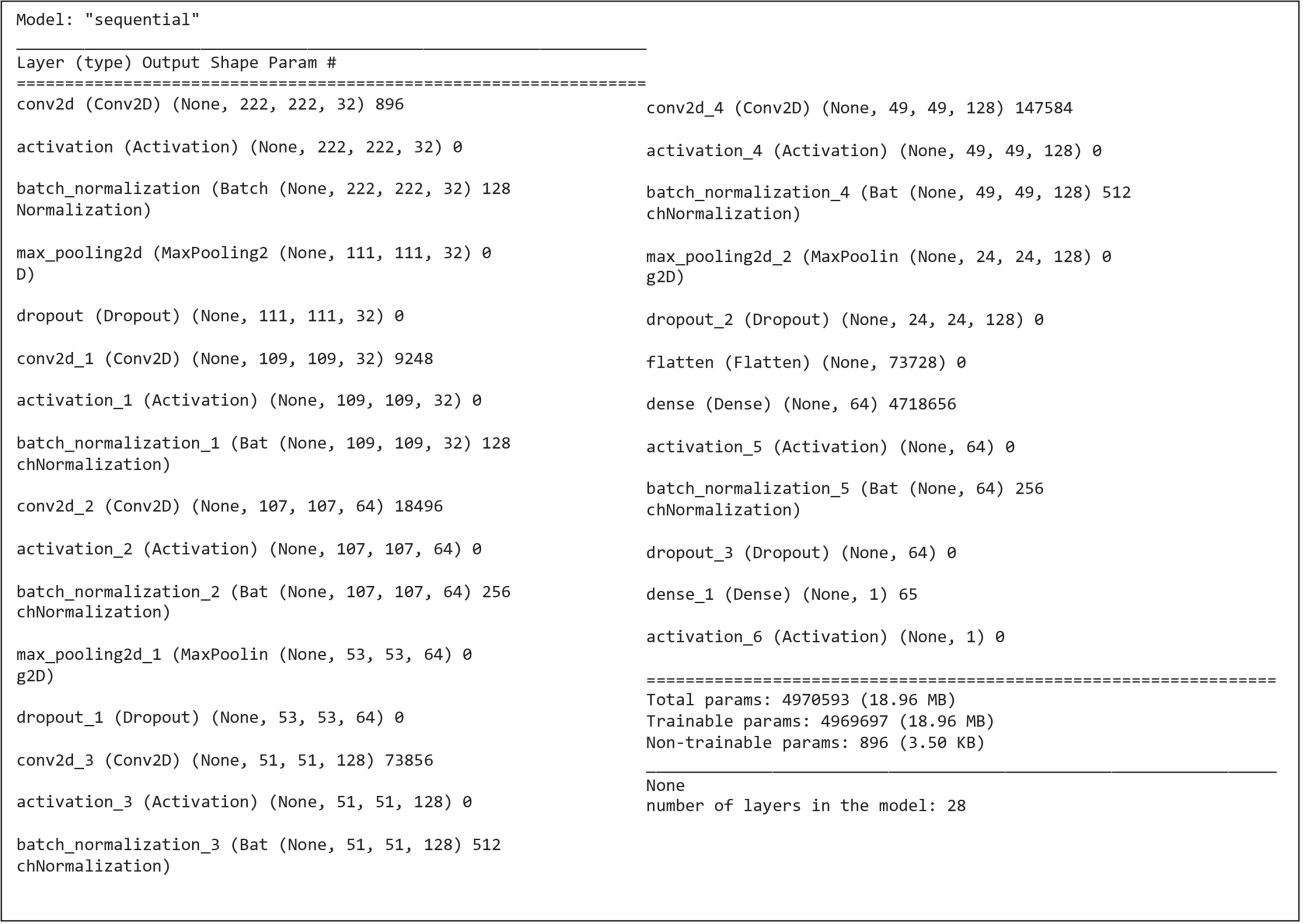
Detailed implementation of the model

The developed DRA model is designed for analyzing panoramic dental radiographs. This custom-built model features six convolutional layers, complemented by batch normalization, max pooling, and dropout layers. The input size of 224 × 224 is fed into the convolutional layers, each followed by a ReLU activation function and batch normalization. The resulting feature maps are down-sampled by the max pooling layers before being passed to subsequent convolutional layers. The final feature map from the last max pooling layer is then processed by a flattening layer to stabilize training and enhance generalization. The model leverages batch normalization to optimize performance. The DRA model was tailored by adjusting parameters and studying the importance of each layer to achieve its optimal performance. The detailed architecture of the DRA model, which uses multiple layers for classifying panoramic dental radiographs, is illustrated in Figure 5.

Working principle

The working principle of the DRA model for layer-wise operation is described as follows: (1) The input size of the dental radiograph is 224 × 224 pixels. (2) The convolution layer Conv1 generates a feature map using 32 filters. (3) The model starts with a two-dimensional (2D) convolutional layer with 32 filters of size 3 × 3, applying the ReLU activation function, zero padding to maintain spatial dimensions, and batch normalization to normalize activations. (4) A MaxPooling2D layer with a pool size of (3,3) reduces spatial dimensions, while a dropout layer with a rate of 0.25 helps control overfitting. (5) The model then includes two additional sets of convolutional layers, each with activation, batch normalization, max pooling, and dropout; the next set uses 64 filters, and the final set uses 128 filters. (6) Following these convolutional layers, a flattening layer converts the three-dimensional output into a one-dimensional vector. (7) A fully connected (dense) layer with ReLU activation, batch normalization, and dropout is introduced to further regularize the model. (8) The output layer consists of a dense layer with neurons equal to the number of classes, using a sigmoid activation function to generate class probabilities.

Evaluation parameters

The efficiency of a proposed classification model is assessed in the classification success report. Various standard performance measures are used to assess the effectiveness of a custom-built model. The measurements include accuracy, precision, recall, and F1-score. In order to observe the true positive (TP), true negative (TN), false positive (FP), and false negative (FN) scores of the binary classification of dental radiographs, confusion matrices are also constructed for each model. The TP score shows how accurately the model classifies the actual teeth with caries instances as teeth with caries. The FP illustrates how the model incorrectly labels teeth with caries as healthy teeth. This part outlines the measures that are used to assess the categorization performance of the model. The confusion matrix performance measures are employed in an experimental study. Equations are used below to compute these matrices. The matrices are created using the confusion matrix parameter, to assess these measures and the requirement of the value count.

Accuracy = (TP + TN)/(TP + TN + FP + FN)

Precision = (TP)/(TP + FP)

Recall = (TP)/(TP + FN)

F1 − score = 2 ∗ (precision ∗ recall)/(precision + recall)

The confusion matrix is the model’s performance measurement. It compares actual and anticipated values as TP, FN, TN, and FN [21].

## Results

The cloud-based platform Google Colab is used to run Python programming. A Windows 11 personal computer equipped with an Intel® Core™ i7 CPU and 16 GB of RAM is used to execute the complete code. Google Colab is used for the model and feature selection training, testing, and validation processes. Experiment analysis is performed three times on the three different datasets mentioned earlier. Binary classification is performed on the pediatric dental radiographs dataset, with two classes, as mentioned: “healthy teeth” and “teeth with caries.” Teeth with restoration and without restoration are the two classes used to evaluate the model’s performance for binary classification using the UFBA dental radiographs dataset. Further region-based anomaly detection is performed on Tuft’s dental panoramic dataset. Typically, “periapical region-based anomaly” and “no anomaly” are the classes used for the evaluation of the model. The result of the developed model for three different datasets is showcased as follows:

Results of the pediatric dental radiograph binary classification: healthy teeth and teeth with caries

**Figure 6:**
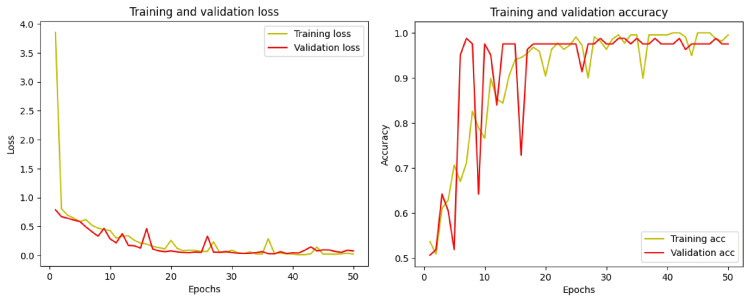
Plot of the training and validation accuracy and loss at each epoch

The training loss, validation loss, and accuracy assessments of the proposed DRA model using pediatric dental radiographs are illustrated in Figure 6. The model’s accuracy, depicted in the second graph, increases over the course of the epochs. While the training loss consistently decreased from 0 to 50 iterations, the validation loss also showed a reduction with each epoch, as demonstrated in the figure.

**Figure 7:**
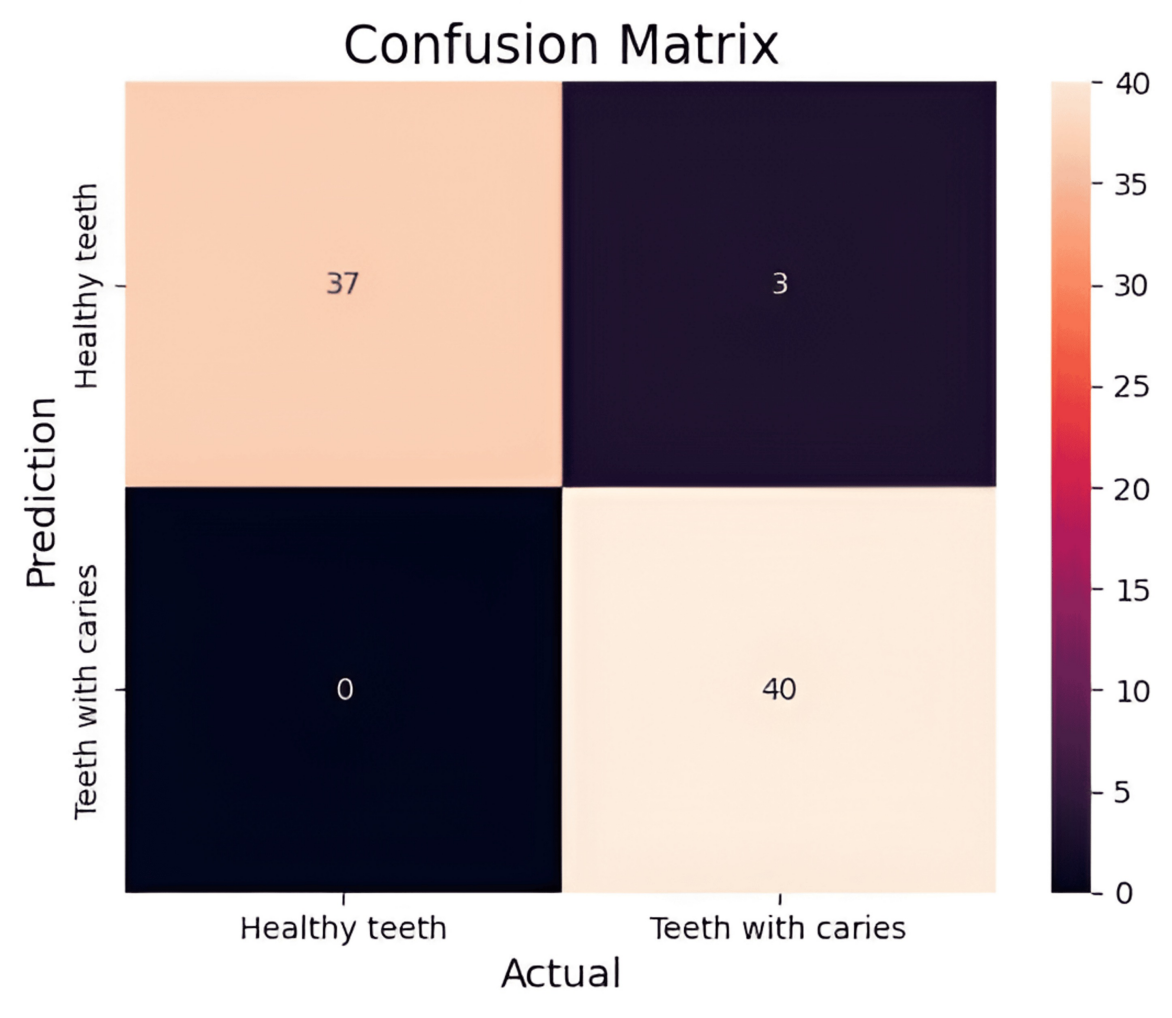
Confusion matrix of model

The model is tested using both unprocessed and preprocessed panoramic dental radiograph datasets. Table 1 shows that using a preprocessing strategy improves results and achieves an accuracy of 97.01%. The binary confusion matrix on the pediatric dental radiograph dataset, which predicts healthy teeth and teeth with caries using the proposed model, is shown in Figure 7. The result shows that out of 80 images, the test data model misclassified only four images.

**Table 1:** Classification result of the model DRA, dental radiograph analysis

Model	Accuracy	Precision	Recall	F1 score
DRA model with unpreprocessed data	0.932	0.8934	0.9401	0.9014
DRA with preprocessed data	0.9701	0.9012	0.9977	0.9423

**Figure 8:**
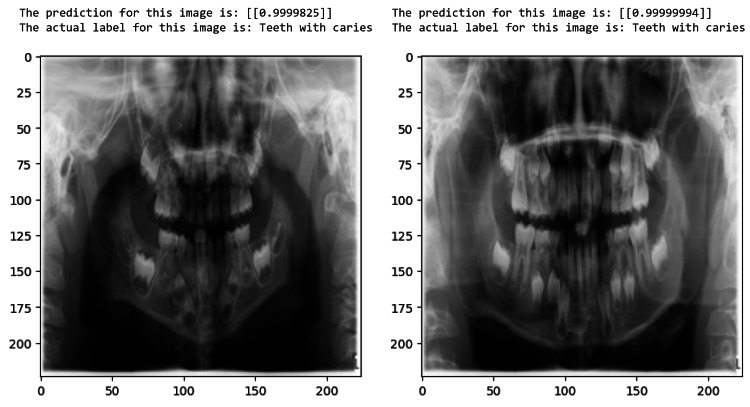
Teeth with caries predicted by the model

**Figure 9:**
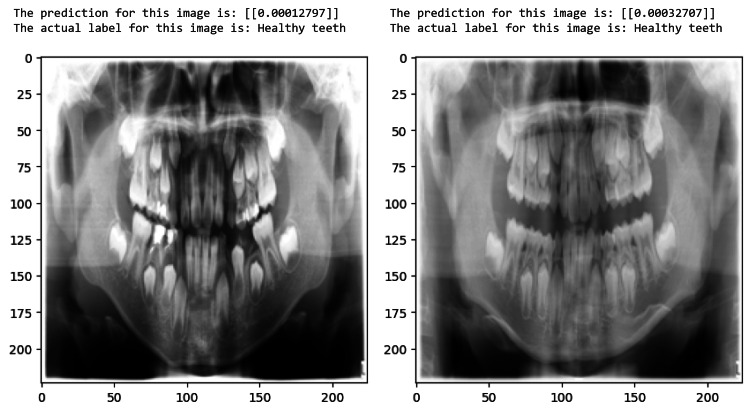
Healthy teeth predicted by the model

**Figure 10:**
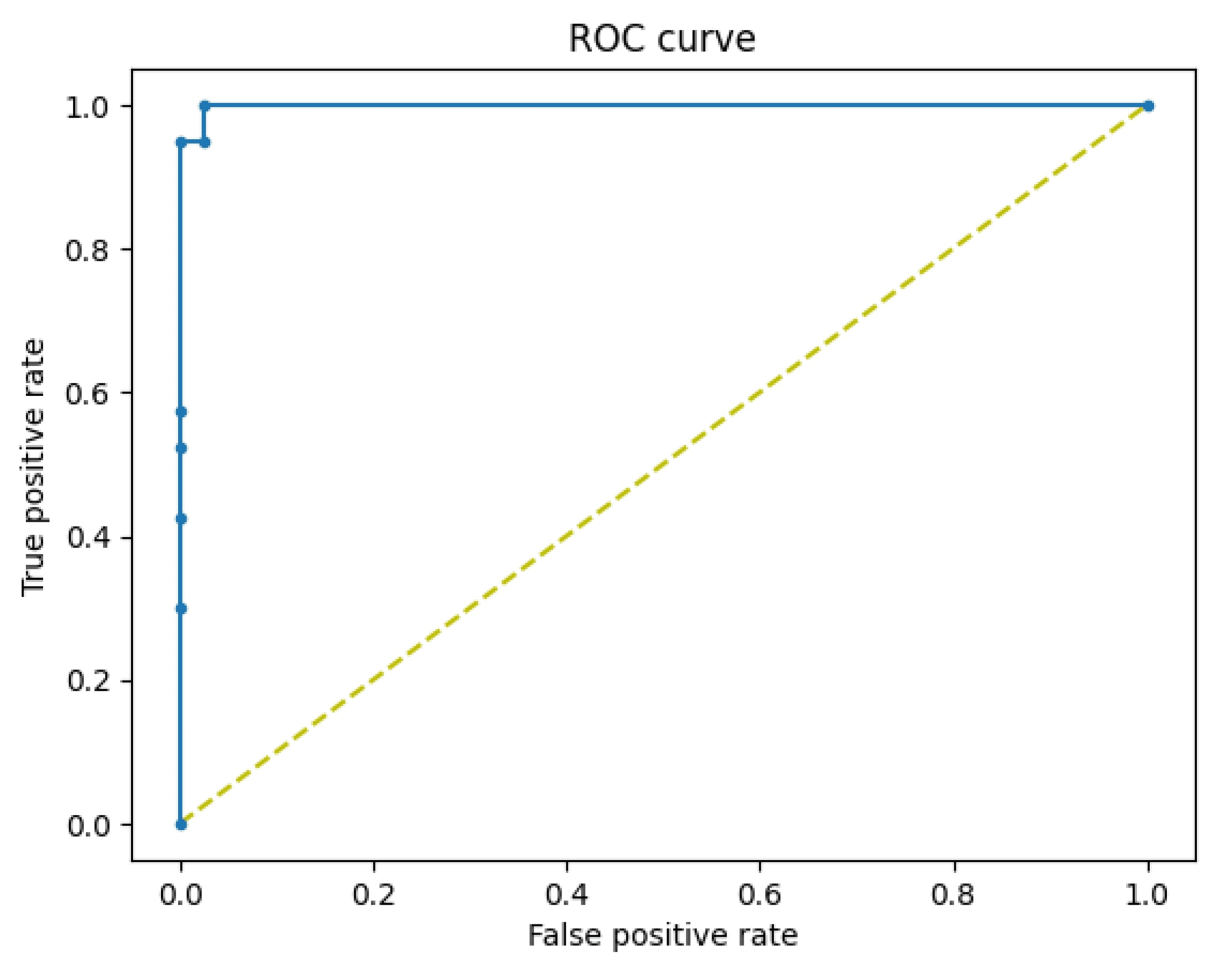
ROC curve of the proposed DRA model (healthy teeth vs. teeth with caries) DRA, dental radiograph analysis; ROC, receiver operating characteristic

The proposed model can identify test samples and determine if teeth are healthy or with caries, as demonstrated in Figure 8 and Figure 9. The ROC curve of a model with pediatric dental radiographs is shown in Figure 10.

Results of the UFBA dental radiograph binary classification: teeth with restoration and teeth without restoration

**Figure 11:**
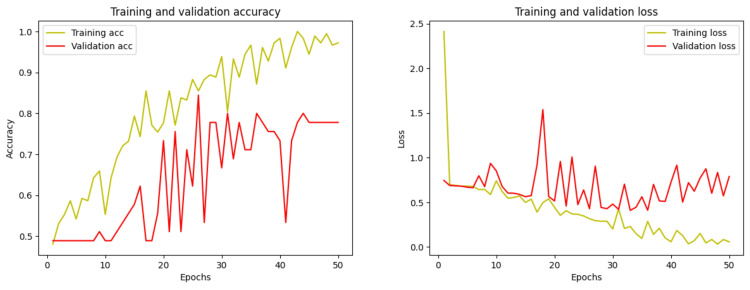
Plot of the training and validation accuracy and loss at each epoch

**Figure 12:**
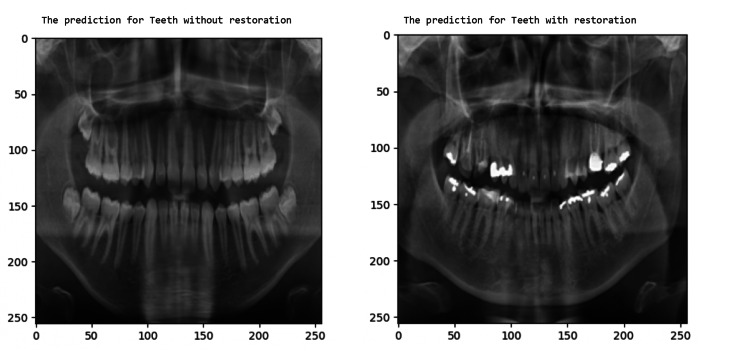
Dental radiographs with restoration predicted by the model

This experiment uses the UFBA dental dataset, and this dataset publishes panoramic dental radiographs in which teeth are with and without restorations; these two categories were used for the binary classification in this experiment. The model predicts the panoramic dental radiographs with and without restorations with the result parameters shown in Table 2. The graph of training and validation accuracy is shown in Figure 11, which shows that the accuracy for this dataset drops to 81.63%. Figure 12 shows the model’s TP sample prediction for binary classification of dental radiographs of the teeth with and without restoration.

**Table 2:** Classification results of different datasets UFBA, Universidade Federal da Bahia

Model	Accuracy	Precision	Recall	F1 score
UFBA	0.8163	0.8181	0.9	0.857
Tuft	0.7753	0.6363	0.875	0.7367

Results of Tuft dental radiograph binary classification: teeth with no anomaly and teeth with periapical region-based anomaly

**Figure 13:**
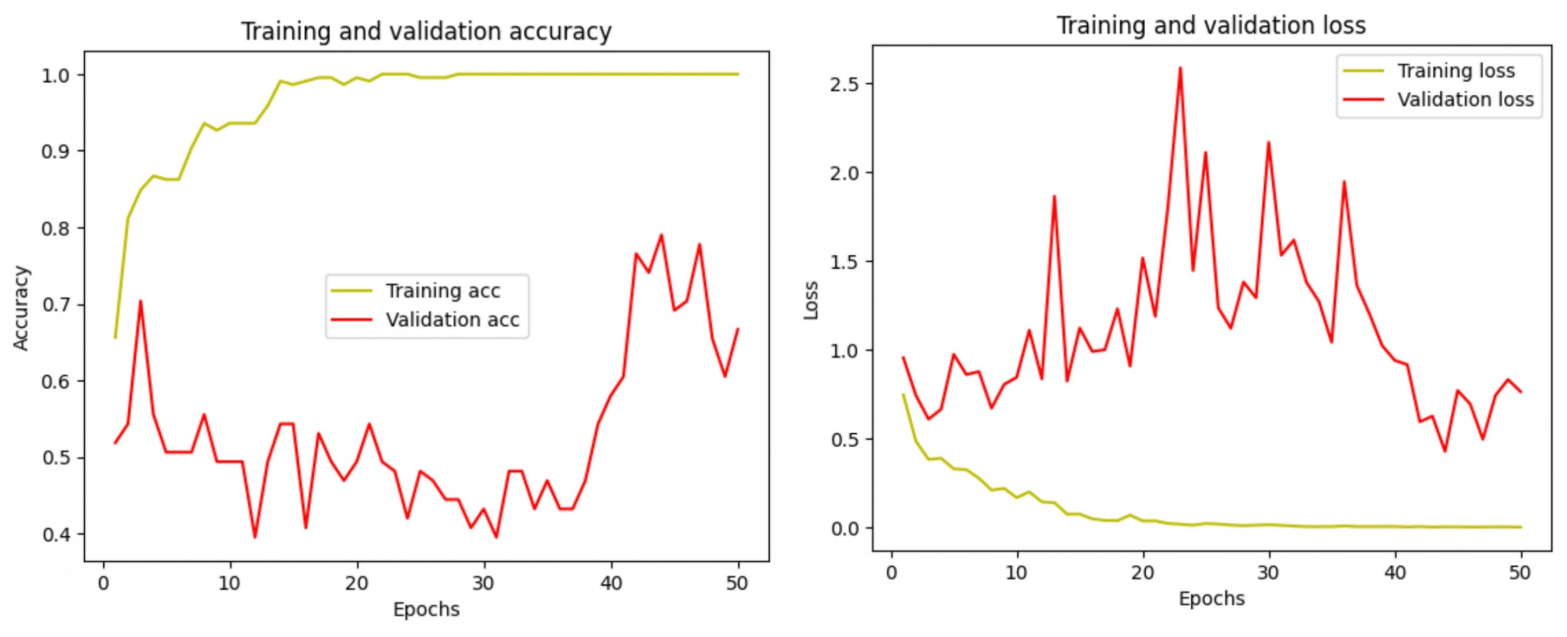
Plot of the training and validation accuracy and loss at each epoch

**Figure 14:**
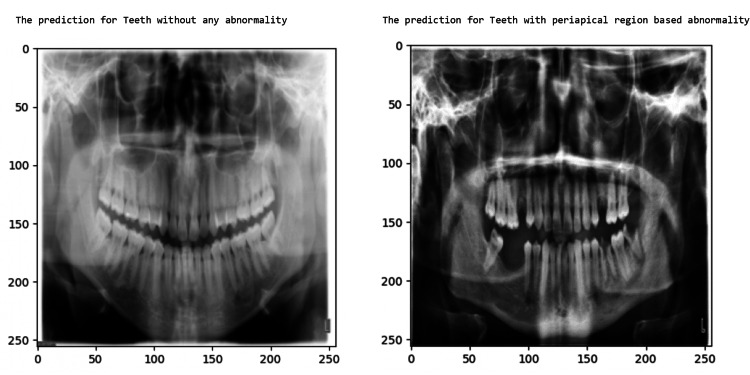
Dental radiograph with periapical region-based abnormality predicted by the model

In this experiment, the Tuft dental dataset is used, which publishes panoramic dental radiographs in which the teeth have periapical region-based abnormalities and without abnormalities; these two categories are used for binary classification. The model predicts the panoramic dental radiographs with periapical region-based abnormality and without abnormality. The obtained result is shown in Table 2, and the training and validation accuracy is shown in Figure 13. Figure 14 shows the model’s TP sample prediction for binary classification of dental radiograph teeth with periapical region-based abnormality and without abnormality.

Based on the above three experiments, it is observed that various factors affect the custom-built CNN model, and the accuracy varies for all of the abovementioned datasets. One of the major reasons is that different datasets may differ in terms of quality, resolution, lighting conditions, patient demographics, and the type of dental anomaly present. These variables majorly impact the model’s performance, as it might not generalize correctly to unforeseen variances. The custom-built model’s architecture complexity, as well as the hyperparameter utilization during training, impacts the model’s performance.

Comparison with different CNN models

**Figure 15:**
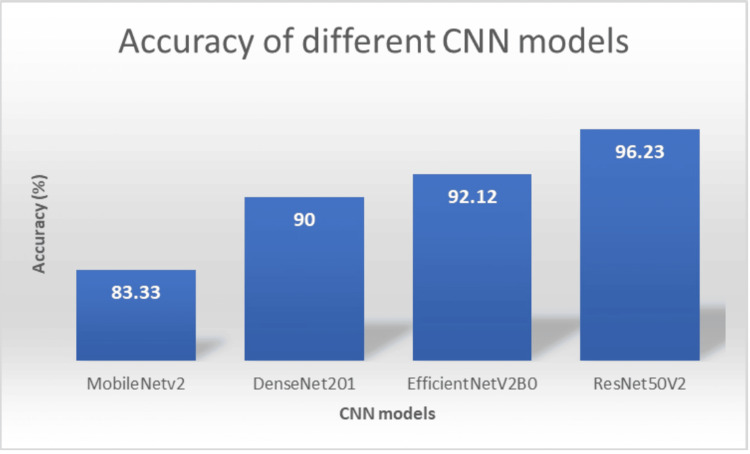
Overall accuracy of various CNN models CNN, convolutional neural network

Accuracy is an important parameter for determining the overall model’s performance. Our suggested model with 97.01% accuracy outperforms all the existing models, i.e., MobileNetv2 (83.33), DenseNet201 (90.00), EfficientNetV2B0 (92.12), and ResNet50v2 (96.23). Figure 15 shows the accuracy comparison, demonstrating the proposed model’s higher performance. This experiment uses the same data sets to measure the performance of the pre-trained models.

## Discussion

The research findings demonstrate how DL can be very beneficial for detecting caries. Our model produced outstanding results without the need for pre-trained models, which are computationally intensive. The ROC and accuracy scores were over 97%. The model accurately detected a considerable number of dental caries cases. The model accurately identifies caries and avoids making erroneous predictions, as evidenced by its highest validation accuracy. Independently trained models without pre-trained weights can achieve significant accuracy in diagnosing caries. This research work analyzed the effectiveness of a custom-built CNN architecture with minimum convolution and max-pooling layers for the detection of dental caries. Our hypothesis is that a small number of convolutional or feature extraction layers may effectively handle simple radiograph binary classification tasks, such as designing dental caries detection systems.

Comparison with current techniques

Our CNN model outperforms conventional methods for detecting caries. Previous methods relied on pre-trained models trained with transfer learning, which required a lot of computing power. Our model outperformed other methods in terms of accuracy. The proposed study achieves 97.01% accuracy in the binary class category for healthy teeth and teeth with caries in the pediatric dental radiograph dataset.

Table 3 presents the performance characteristics of state-of-the-art approaches compared to the proposed DRA model. It was observed that using pre-trained models did not enhance model robustness [29]. In contrast, the proposed model significantly improved its effectiveness and robustness. The custom-built model outperforms existing models in terms of training time and requires fewer trainable parameters.

**Table 3:** Comparison of the proposed method with existing techniques in the literature CNN, convolutional neural network; DRA, dental radiograph analysis; IoU, intersection over union; LSTM, long short-term memory; SVM, support vector machine

Model image	Image type	Primary performance metrics and values
MobileNetv2 [9]	Panoramic	Accuracy: 87.00
Modified ResNet18 [10]	Periapical	F1 score: 82.90
Hybrid neural network [22]	Bitwing	Accuracy: 90.00
CNN-LSTM [23]	Periapical	Accuracy: 96.00
CNN-SVM [24]	Panoramic	Accuracy: 93.58
CNN and capsule classifier [25]	Panoramic	Accuracy: 86.05
Swin transformer [26]	Panoramic	Accuracy: 85.57
Faster R-CNN [27]	Periapical	IoU: 71.59
Fast R-CNN [28]	Panoramic	Accuracy: 90
Proposed DRA model	Panoramic	Accuracy: 97.01

Integration with clinical practice

DL models, such as CNN, have the potential to significantly impact clinical practice for caries detection, particularly in economically backward areas. This DRA model can help radiologists detect dental caries more quickly and correctly by analyzing panoramic dental radiographs. This could aid in the early prevention of dental diseases. Early and precise diagnosis of caries can result in the implementation of suitable preventive and conservative measures, which can reduce the cost of healthcare.

Limitations

Several factors contribute to variations in the output results of the experiments, including data preparation methods such as resizing, which impact model performance. Different preprocessing strategies may be more suitable for some datasets than others, leading to discrepancies in accuracy. Variations in training strategies, including optimization methods, learning rates, and the number of epochs, also affect final model performance. Additionally, since the model’s weights are typically randomized, addressing these challenges requires a thorough analysis of the dataset, fine-tuning of the model’s architecture, and adjustment of hyperparameters. Ongoing research in these areas could eventually be applied to real-world scenarios. Proper preprocessing techniques and strategies for handling imbalanced datasets should also be considered. Evaluating various datasets can provide a deeper understanding of the model’s performance and generalization potential. To improve accuracy and robustness, incorporating advanced image processing techniques and increasing the dataset's sample size would be beneficial.

Future scope

The future plan is to enhance the model’s efficiency by incorporating additional datasets featuring new panoramic dental radiographs. Utilizing more radiographs from real-world clinical settings rather than relying solely on standardized online databases will enable the model to better handle variations in image quality, patient positioning, and different dental equipment. This approach aims to improve the model's ability to analyze panoramic dental radiographs and provide more comprehensive support for dental care.

## Conclusions

This work describes a DL-based DRA model for the binary classification of pediatric panoramic dental radiographs, aimed at distinguishing between healthy teeth and those with caries. The same model was also applied to the UFBA-UESC dataset to classify teeth with or without restoration and to the Tuft dental dataset for detecting periapical region-based anomalies. The study demonstrates the model’s effectiveness in achieving accurate binary classification results through rigorous experimentation with diverse datasets. Training the custom-built DRA model without pre-trained weights required fewer processing resources and achieved a commendable accuracy of 97.01% for caries detection. Performance was analyzed using visualization measures such as the ROC curve, validation accuracy, precision, recall, and F1 score. The model’s efficient performance can reduce the risk of human error associated with subjective evaluations during diagnosis. Early and precise detection of caries facilitates the implementation of appropriate preventive and conservative interventions, ultimately reducing healthcare costs.
